# Boundary aware microscopic hyperspectral pathology image segmentation network guided by information entropy weight

**DOI:** 10.3389/fonc.2025.1549544

**Published:** 2025-03-27

**Authors:** Xueying Cao, Hongmin Gao, Ting Qin, Min Zhu, Ping Zhang, Peipei Xu

**Affiliations:** ^1^ College of Computer Science and Software Engineering, Hohai University, Nanjing, China; ^2^ Department of Hematology, Nanjing Drum Tower Hospital Clinical College of Nanjing Medical University, Nanjing, China; ^3^ School of Information Management & Engineering, Shanghai University of Finance and Economics, Shanghai, China; ^4^ College of International Exchange, Nanjing Normal University of Special Education, Nanjing, China; ^5^ Department of Hematology, Nanjing Drum Tower Hospital, Affiliated Hospital of Medical School, Nanjing University, Nanjing, China

**Keywords:** microscopic hyperspectral image, boundary-aware, information entropy, attention mechanism, multi-scale

## Abstract

**Introduction:**

Accurate segmentation of lesion tissues in medical microscopic hyperspectral pathological images is crucial for enhancing early tumor diagnosis and improving patient prognosis. However, the complex structure and indistinct boundaries of lesion tissues present significant challenges in achieving precise segmentation.

**Methods:**

To address these challenges, we propose a novel method named BE-Net. It employs multi-scale strategy and edge operators to capture fine edge details, while incorporating information entropy to construct attention mechanisms that further strengthen the representation of relevant features. Specifically, we first propose a Laplacian of Gaussian operator convolution boundary feature extraction block, which encodes feature gradient information through the improved edge detection operators and emphasizes relevant boundary channel weights based on channel information entropy weighting. We further designed a grouped multi-scale edge feature extraction module to optimize the fusion process between the encoder and decoder, with the goal of optimize boundary details and emphasizing relevant channel representations. Finally, we propose a multi-scale spatial boundary feature extraction block to guide the model in emphasizing the most important spatial locations and boundary regions.

**Result:**

We evaluate BE-Net on medical microscopic hyperspectral pathological image datasets of gastric intraepithelial neoplasia and gastric mucosal intestinal metaplasia. Experimental results demonstrate that BE-Net outperforms other state-of-the-art segmentation methods in terms of accuracy and boundary preservation.

**Discussion:**

This advance has significant implications for the field of MHSIs segmentation. Our code is freely available at https://github.com/sharycao/BE-NET.

## 1 Introduction

Microscopic hyperspectral pathological images (MHSIs) contain both high spatial resolution and high spectral resolution (1). Compared to traditional RGB images, MHSIs not only reflect the spatial structure of biological cells or tissues but also reveal their molecular and functional information by spectral bands (2, 3). By analyzing spatial-spectral feature information, it can help identify subtle tumor characteristics that human experts may not immediately detect, as well as edge features in infiltrating areas, providing valuable insights for early and accurate diagnosis. However, the varying shapes, sizes, and boundaries of biological tissues, coupled with the redundant spectral features in MHSIs, present significant challenges in developing robust and precise segmentation methods for these images.

To address the aforementioned challenges, researchers have developed a large number of encoder-decoder networks, based on convolutional neural network (CNN), such as U-Net (4), U-net++ (5). Owing to the powerful hierarchical feature extraction capabilities of CNN, these methods have delivered outstanding segmentation results (6). However, the weight-sharing mechanism of CNN result in the same processing being applied to all positions on the feature map when extracting features, which may prevent the network from focusing on key features (7, 8). Moreover, since the convolutional kernels are optimized through random initialization, they often lack specificity in capturing boundary features (9). To mitigate the aforementioned issues, numerous channel and spatial attention mechanisms are designed to emphasize important features which highly relevant to the task (10, 11). Currently, these methods have been integrated into various encoder-decoder networks, such as CA-Net (12), Att-UNet (13), TransUnet (14) and MissFormer (15). The aforementioned method has achieved significant success by adaptively computing the similarity or correlation between features. However, they overlook the uncertainty of the features themselves (12, 13), which can lead to susceptibility to noise interference when dealing with complex backgrounds. This makes it a challenging task to further enhance the model’s ability to capture key features. Furthermore, some studies have introduced edge detection operators to encode gradient information between features, aiming to capture the edge details within the images more effectively (7, 16). However, due to the influence of the phenomena of “different objects with the same spectral” and “the same object with different spectral “ in MHSIs, using traditional edge operators to capture edge information channel by channel and directly overlaying them introduces a large amount of redundancy and noise, thereby affecting the final segmentation performance.

Information entropy (17) is a measure used to quantify the amount of information of a random variable (18). Through information entropy, researchers can comprehensively understand of an important random variable (19), and the comprehensiveness is one of the most significant factors for data-driven multiscale analysis of complex data (20). By evaluating features through information entropy and using the assessment results to guide the model to focus on the most relevant regions, the model’s ability to effectively extract features can be further enhanced. However, existing MHSIs segmentation methods have overlooked research on assigning weights to spectral and spatial features based on entropy weighting. Therefore, researching information entropy-weighted attention mechanisms to guide the model more stably focus on key features is of great significance for further improving segmentation accuracy.

In summary, considering the importance of detail and boundary features in the accurate segmentation of MHSIs, and the ability of information entropy weighting to guide the model in capturing key information, a boundary aware MHSI segmentation network guided by information entropy weight (BE-Net) is proposed. Specifically, we first design Laplacian of Gaussian operator-convolution boundary feature extraction block (LCB). LCB integrates Laplacian of Gaussian operator (LoG) with convolution operations, facilitating the extraction of more efficient and robust gradient features. Subsequently, channel entropy-weighted attention (CEA) is employed to further enhance the representation of relevant edge channels while suppressing redundant information. This novel approach fully capitalizes on the strengths of both the LoG operator and CNN, allowing the model to more effectively adapt to the diversity and complexity of MHSIs. In addition, we developed a group multi-scale boundary feature extraction block (GMB). This block enhances the model’s focus on detailed features by utilizing group convolution and multi-scale feature extraction and employs CEA to further enhance the boundary contours of the segmentation area. Finally, we designed a multi-scale spatial boundary feature extraction block (MSB). This module consists of a hierarchical multi-scale feature extraction block (HMB), a multi-scale pooling fusion block (MPB), and spatial entropy-weighted attention (SEA). HMB helps to comprehensively capture the characteristics of biological tissues of different sizes and shapes, while MPB emphasizes important features through max pooling at different scales. The SEA enhances the high-entropy features which may be highly correlated with edge height, for further delineating the edges of biological tissues. Our contributions can be summarized as follows:

1. This paper introduces an information entropy-guided boundary-aware network, which captures edge and detailed information in MHSIs to further enhance channel and spatial feature representations, thereby improving segmentation accuracy. To the best of our knowledge, this is the first study to apply information entropy to the task of MHSI segmentation.
2. This paper develops three novel modules including LCB, GMB, and MSB. LCB employs more flexible edge detectors designed based on LoG and CNN to obtain boundary information. GMB captures the boundary details and fine representations of biological tissues through grouped multi-scale feature extraction. Both LCB and GMB leverage channel entropy-weighted attention to further guide the extracted features, allowing the model to focus on critical channels. MSB utilizes HMB to further enrich feature information, while MPB enhances the contrast between the segmentation target and surrounding regions. In addition, SEA directs the model to focus on high-entropy features, further strengthening the ability to extract edge features.
3. The proposed network model was evaluated on gastric mucosal intestinal metaplasia (IM) and gastric intraepithelial neoplasia (GIN) microscopic hyperspectral pathological images, and a large number of experimental results showed that the method is superior to other advanced methods.

## 2 Related work

### 2.1 Traditional edge detectors

In early studies, researchers used gradient operators [Robert operator (21), Sobel operator (22), and Prewitt operator (23)] to detect boundary information. For obtaining refined boundary and mitigate the impact of noise on edge detectors, some methods introduce the Laplacian of Gaussian operator (LoG) into edge detect task (24). These methods initially utilize a Gaussian operator to smooth the original image, reducing isolated noise points and smaller structural elements. Subsequently, edge detection is carried out using the Laplacian operator. Additionally, considering that MHSIs contain both rich spatial and spectral information, some studies utilized spectral divergence (25) and spectral angle measurement (26) into the existing edge detection operators. By combining spatial and spectral features, detection accuracy and adaptability in complex scenes can be enhanced. However, most of the aforementioned methods are sensitive to noise and rely on simple linear structures, which makes it difficult to capture key pathological features from the complex spatial and spectral characteristics of MHSIs.

### 2.2 Information entropy

Information entropy can reasonably quantify the statistical characteristics of information (27), and it, along with its variants, widely applied in fields such as data dimensionality reduction, feature selection, and data compression. For example, He et al. (28) designed a novel entropy-based principal component analysis method for automatic dimensionality reduction of electrocardiogram (ECG) signals. Shi et al. (29) proposed a sparse kernel entropy component analysis algorithm to address the challenge of small-sample high-dimensional biomedical data. Xu et al. (27) introduced a fuzzy data feature selection method based on neighborhood rough set and local composite entropy, further enhancing the ability of information entropy to describe uncertainty. Huang et al. (30) proposed a feature selection method based on conditional entropy. In the field of data compression, entropy coding re-encodes images based on probabilities to reduce redundant information in the data (31). In summary, as a measure that helps researchers comprehensively understand features, information entropy can assist models in identifying task-relevant features, thereby enhancing model performance across various tasks. Thus, incorporating information entropy into the MHSI segmentation task holds significant potential for further improving the model’s ability to learn boundary information.

### 2.3 Attention mechanism

The attention mechanism mimics the dynamic selection process of the human visual system (32), adaptively weighting features based on their importance in the input (33). In recent years, the attention mechanism has played an increasingly important role in fields such as image classification (34), semantic segmentation (35), and object detection (36). Among these, channel attention and spatial attention are two of the most important attention mechanisms.

Channel attention assigns different weights to different channels, which can be regarded as a process of object selection (33). In 2017, Hu et al. (8) introduced SE-Net, the first channel attention mechanism, which implements channel attention in two stages: Squeeze and Excitation. Since then, numerous researchers have built upon this foundation, making improvements and achieving remarkable results (37, 38). Inspired by SE-Net, Hu et al. (39) designed a spatial attention mechanism incorporating gather and extraction operations to capture spatial context information. Though spatial attention, important spatial locations in the feature map are emphasized while irrelevant regions are suppressed. To further capture global context, several self-attention models, such as Non-local (11), Vision Transformer (ViT) (40), and MissFormer (15) have been proposed. Additionally, some researchers have combined spatial attention with channel attention, weighting the feature maps from different dimensions to achieve more comprehensive and refined feature extraction.

Thanks to the great success of the attention mechanism, researchers have introduced them into medical image segmentation tasks, using them to emphasize key information. For example, Huang et al. (41) proposed a channel prior convolutional attention segmentation network to tackle the issue of low contrast in medical images. Chen et al. (42) proposed a multi-scale channel attention mechanism to improve the accuracy of medical ultrasound image segmentation. He et al. (43) developed a boundary-guided filtering network for medical image segmentation. This network uses deep semantics to guide shallow features and combines channel attention, spatial attention, and boundary guided filters to capture structural information within the features. Yu et al. (44) built a boundary-aware mechanism based on gradient convolution, utilizing a pooling method in the channel dimension to obtain the relationship weights between multiple channel feature.

## 3 Methods

**Figure 1:**
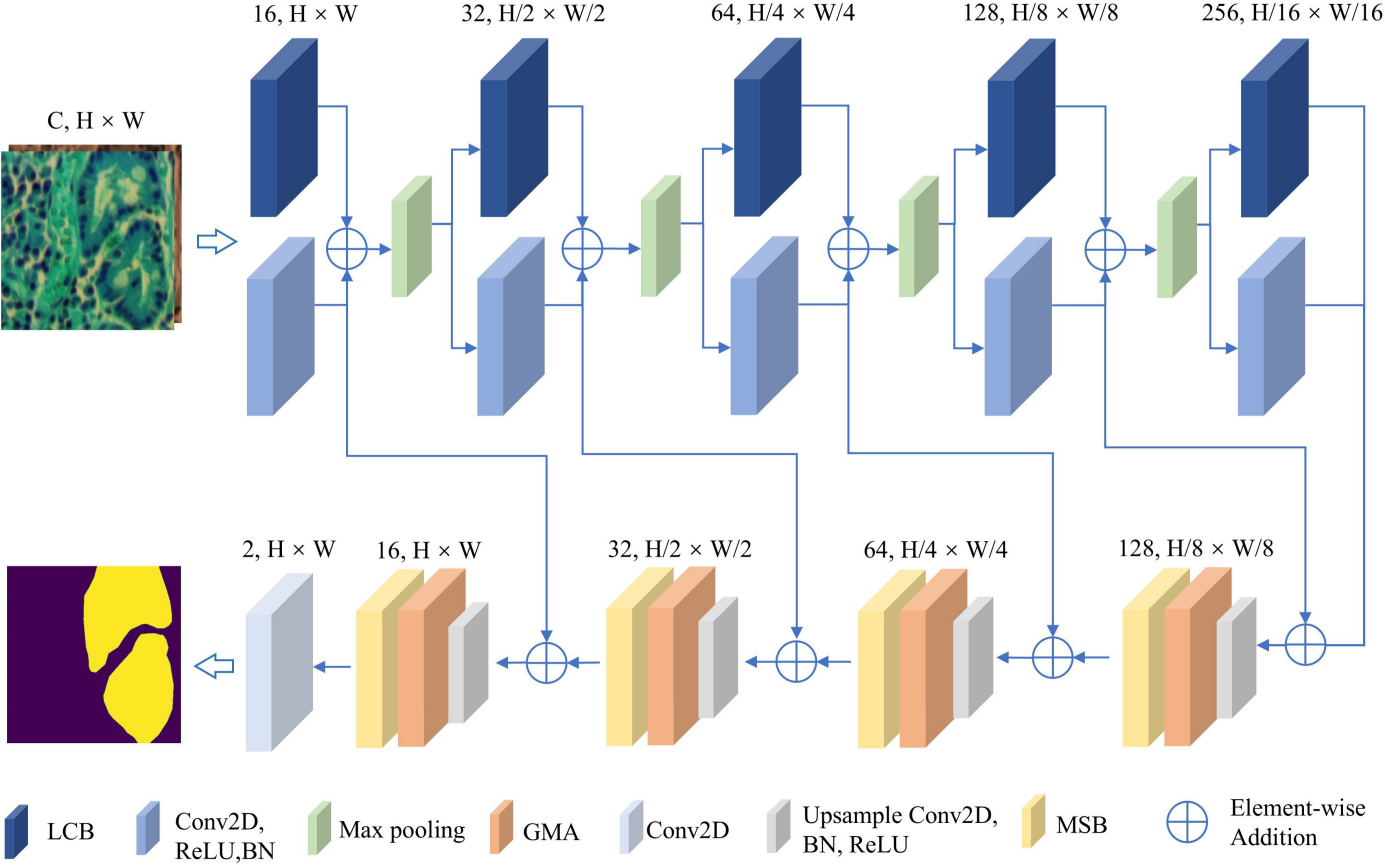
Overall architecture of BE-Net.

As shown in **Figure 1**, the proposed BE-Net is an encoder-decoder network built upon the U-Net architecture. In the encoder, we designed LCB to delve deeper into the intricate boundary information of tissue. Specifically, in this module, the proposed LoGC is employed to extract edge information, while the CEA is responsible for emphasizing which edge information needs to be prioritized. Both components play crucial roles in the overall process. In the decoder, we designed the GMB and MSB to further extract and emphasize the key features of the segmentation task. Specifically, the shallow features are fused with deep semantics through the GMB. The resulting fused features are then passed to the MSB, where a HMB is used to further enrich the extracted feature information. Subsequently, MPB and SEA are applied to further enhance detailed features and refine boundary information.

In addition, to reduce the computational cost, we reduced the number of channels at each level in the U-net to one-quarter of the original and applied principal component analysis (PCA) to lower the number of spectral bands in the original MHSIs. We will provide a detailed description of the above modules in the following sections.

### 3.1 Laplacian of Gaussian convolution operator boundary feature extraction block

**Figure 2:**
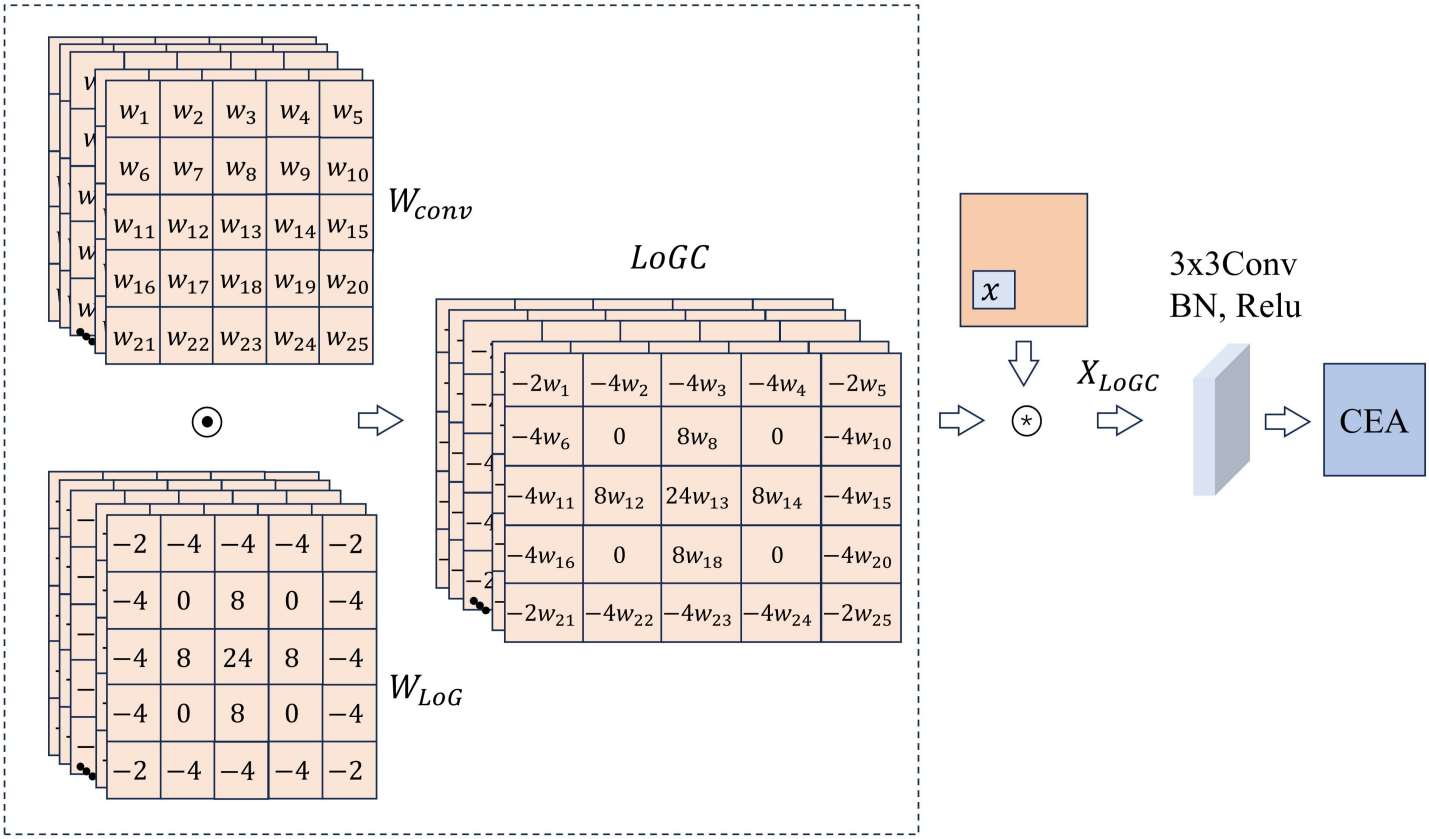
Overall architecture of LCB.

Fully extracting and utilizing edge information is essential for improving the accuracy of image segmentation. To efficiently explore the edge information of the tissue in MHSIs, we designed LCB module, which consists of a LoGC, a 3x3 convolution, and a CEA. LoGC is designed by multiplying the LoG with convolutional kernels pixel by pixel. Its aggregate features across the feature map, allowing the model to better learn the trend of feature changes. We show the overall design of the module in **Figure 2**.

The LoG operator consists of two components: the Gaussian operator, which is used to smooth noise, and the Laplacian operator, which is responsible for detecting edges. Given the presence of a substantial amount of isolated biological tissues in non-target areas of MHSIs, these interferences and redundancies can significantly impact the accuracy of edge detection. Therefore, applying Gaussian filtering before capturing edge features can reduce the interference caused by the aforementioned tissues, thereby achieving better segmentation results. Assuming p and q are the spatial pixel coordinates of the MHSI. The above process can be represented as Equations 1-3:


(1) $$G\left(p,q,\sigma \right)=\;-\frac{1}{2\pi \sigma }e^{-\frac{p^{2}+q^{2}}{2\sigma^{2}}}$$

(2) $$\nabla L\left(p,q\right)=\frac{\partial L}{\partial p^{2}}+\frac{\partial L}{\partial q^{2}}$$

(3) $$LoG\left(p,q,\sigma \right)=\;\frac{\partial G}{\partial p^{2}}+\frac{\partial G}{\partial q^{2}}=\frac{1}{\pi \sigma^{4}}\left(\frac{p^{2}+q^{2}}{2\sigma^{2}}-1\right)e^{-\frac{p^{2}+q^{2}}{2\sigma^{2}}}\;$$

where G(p,q,σ) is the Gaussian operator formula, σ is the scale factor, ∇L(p,q) is the Laplace operator and LoG(p,q,σ) refers to the LoG operator. Since the discrete representation of MHSIs, Equation 3 is discretized to approximate the LoG operator used in practical processes. As shown in **Figure 2**, the LoG operator multiply with the weights in the convolution kernel to generate the final 5×5 LoGC operator. The definition of the LoGC operator is presented in Equation 4.


(4) $$X_{LoGC}=\;x⊛\left(w_{Conv}⊙w_{LoG}\right)$$

where x represents the input feature, $w_{Conv}$ represents the convolution kernel weights, $w_{LoG}$ represents the LoG operator matrix, ⊙ denotes the element-wise multiplication, and ⊛ represents the convolution operation.

**Figure 3:**
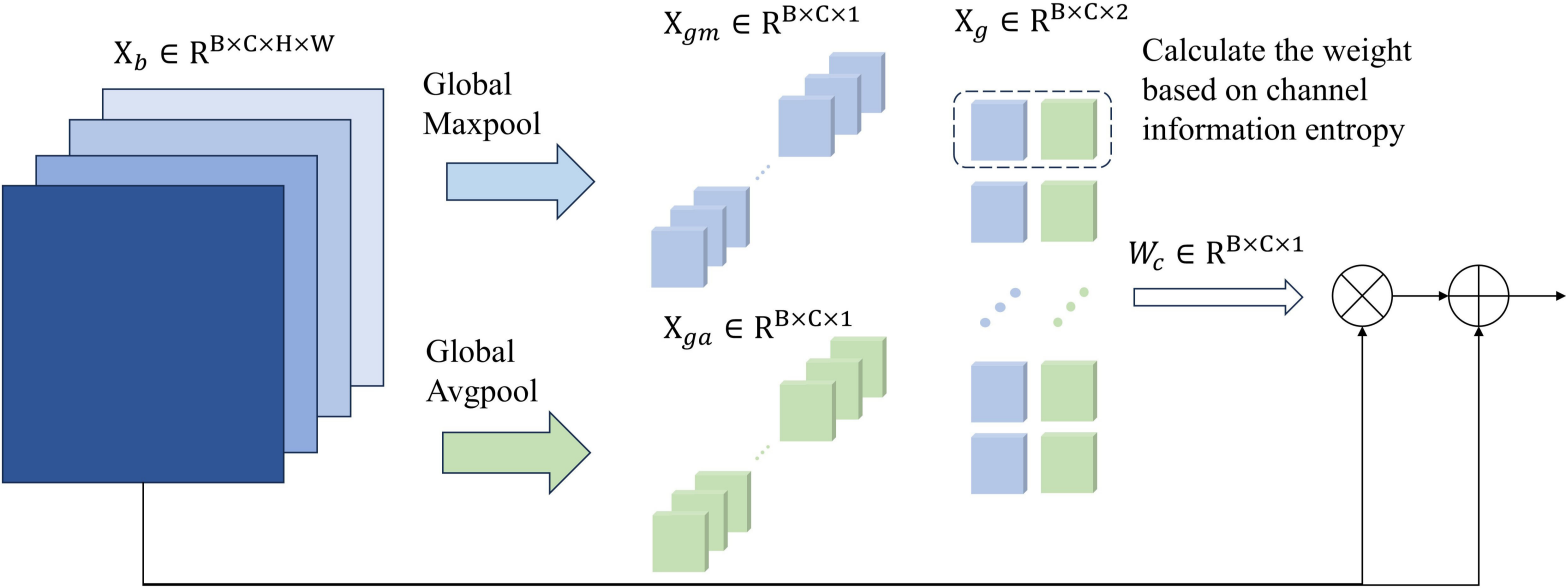
Overall architecture of CEA.


Each channel in the feature cube contains different feature information, and these features contribute differently to the final segmentation performance. Therefore, CEA is designed to emphasize the channels with higher association with edge features, further improving the accuracy of edge segmentation. Considering the significant variation in feature between edge and non-edge regions (43), the proposed CEA utilizes information entropy to quantify the differences between max pooling and average pooling in each channel layer, thereby highlighting the channel layers with the most prominent feature changes. As shown in **Figure 3**, assume the input feature is $X_{b}\in {\mathbb{R}}^{\text{B}\times \text{C}\times \text{H}\times \text{W}}$ , where B is the batch size, C is the number of channels, and H and W represent the height and width of the feature map, respectively. The CEA first applies global max pooling and global average pooling to $X_{b}$ , generating $X_{gm}\in {\mathbb{R}}^{\text{B}\times \text{C}\times 1}$ and $X_{ga}\in {\mathbb{R}}^{\text{B}\times \text{C}\times 1}$ . Then concatenate $X_{gm}$ with $X_{ga}$ to obtain $X_{g}\in {\mathbb{R}}^{\text{B}\times \text{C}\times 2}$ , and calculate each channel’s information entropy in $X_{g}$ . Next, the channel weights of the feature map are reassigned by calculating the ratio between the information entropy of each channel and the maximum possible entropy. This process can be expressed using Equations 5–7.

(5) $$H\left(x_{g}^{c}\right)=-\sum_{i=1}^{N}o\left(x_{g}^{i}\right)\text{log}\left(o\left(x_{g}^{i}\right)\right)$$

(6) $$H_{max}=log\left(N\right)$$

(7) $$W_{c}=1-\frac{H\left(x_{g}^{c}\right)}{H_{max}}$$

where $H\left(x_{g}^{c}\right)$ represents the entropy of c -th channel in $X_{g}$ , $o\left(x_{g}^{i}\right)$ denotes the probability distribution corresponding to the value $x_{g}^{i}$ (in this work, the feature probability is calculated based on the softmax function), and N is the number of elements in the dimension used for entropy calculation (which is 2 in this paper). $H_{max}$ represents the max information entropy value, which occurs in the case of the max and average feature values are equal. Subsequently, CEA assigns greater weights to the channels with smaller information entropy. This is because channels with lower information entropy indicate, on one hand, higher stability and predictability, and on the other hand, a greater disparity between the channel’s maximum and average values, which may suggest the presence of more abundant and representative feature information.

### 3.2 Group multi-scale boundary feature extraction block

As the depth of the network increases, shallow detailed features are gradually lost. To address this issue, skip connections are employed to transfer shallow features to the decoder. These shallow features contain rich geometric characteristics and boundary of tissues, which can help the network preserve detailed information and better reconstruct spatial feature. However, there is a significant gap between the local details contained in these shallow features and the global semantics in the deep features, which makes it challenging to effectively combine the two. To mitigate this issue, we designed GMB. GMB consists of a grouped multi-scale feature extraction module (GM) and CEA. Specifically, GMB enhances the complementarity between shallow and deep features by using specific convolution strategies, while preserving the feature information of each. Subsequently, CEA is applied to further weight the channels containing important feature information, ensuring that critical semantic information is enhanced.

**Figure 4:**
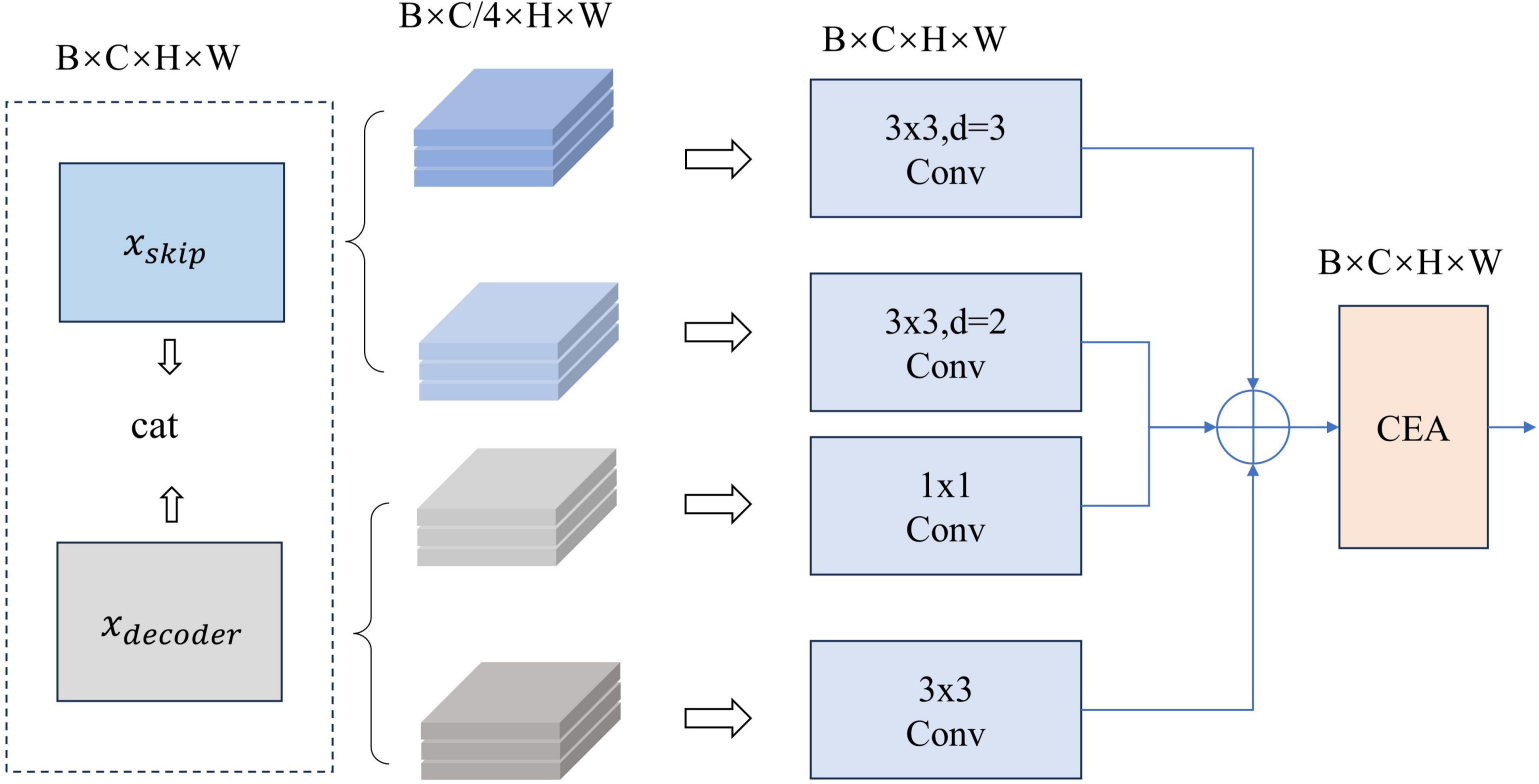
Overall architecture of GMB.


As shown in **Figure 4**, we first stack the shallow features $X_{skip}$ with the deep semantic features $X_{decoder}$ . Then, we evenly divide them into four groups along the channels and apply different convolutions to each group for feature extraction. For shallow features, GMB employs dilated convolutions with kernel size of 3 and dilation rates of 2 and 3, respectively, to capture large-scale structures and broader global context. For deep features, the GMB utilizes 1x1 convolutions and 3x3 convolutions to extract detailed semantic information. By extracting broader semantic features from shallow layers and enhancing local details in deep layers, the semantic gap between them is bridged, and the representational capacity of both is strengthened. These four groups are then fused, and the CEA is applied to highlight channels containing important feature information. The process can be described as Equations 8, 9

(8) $$X_{i}\;=\;split\left(cat\left(X_{skip},\;X_{decoder}\right)\right)$$

(9) $$X=Relu\left(\sum_{i=1}^{4}Conv_{i}\left(X_{i}\right)\right)$$

where cat represents channel-wise concatenation, and split represents dividing the feature map into four parts along the channel axis, with i representing the group number, i∈{1,2,3,4} . $Conv_{i}$ refers to the convolution corresponding to each group. The CEA process is the same as described in Equations 5-7.

### 3.3 Multi-scale spatial boundary feature extraction block

**Figure 5:**
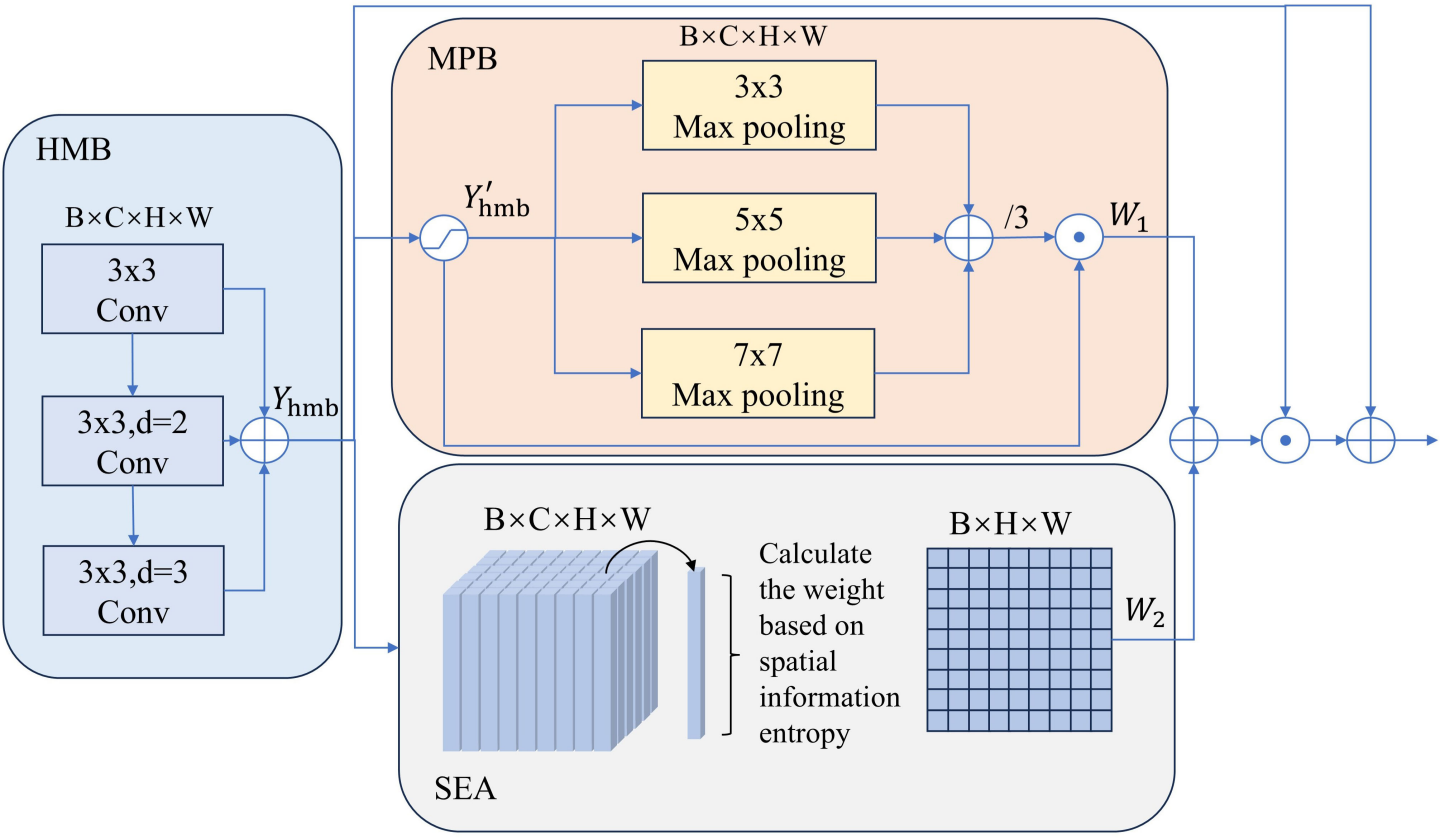
Overall architecture of MSB.

In MHSIs, there is a large amount of spatial background information that is irrelevant to the segmentation task. This redundancy and interference can prevent the model from focusing on the lesion area. In order to solve this problem, MSB is designed. As shown in **Figure 5**, MSB first employs a HMB to obtain a feature pyramid that can reflect spatial information at different scales. Next, using the MPB, irrelevant background features are suppressed while important spatial information is enhanced. Finally, the SEA is employed to focus on the boundary region, further refining the segmentation mask’s boundary.

In HMB, we concatenate convolutions with kernel sizes of 3x3 and dilation rates of 1, 2, and 3, respectively. The feature maps generated by each convolution are then fused to create a multi-scale fusion pyramid, $Y_{hmb}$ . Then, $Y_{hmb}$ is normalized using the sigmoid function to obtain $Y_{hmb}^{'}$ . Next, we design MPB to guide the region of interest. As shown in **Figure 5**, in the MPB, $Y_{hmb}^{'}$ is first processed by three max pooling layers with kernel sizes of 3x3, 5x5 and 7x7 for feature extraction. The three resulting feature maps are then averaged to produce the output. By integrating the max features from context information at multiple scales, more robust spatial weights are obtained that capture spatial intensity variations across scales, enhancing the contrast between the segmentation target and the surrounding areas. In addition, it also mitigates the issue of feature information loss caused by using max pooling alone. Finally, the resulting weight map is multiplied element-wise with $Y_{hmb}^{'}$ to produce $W_{1}$ . The above process can be expressed as Equations 10–12:


(10) $$Y_{hmb}=\;\sum_{i=1}^{3}BN\left(Relu\left(Conv_{i}\left(X_{i-1}\right)\right)\right)$$

(11) $$Y_{hmb}^{'}=Sigmoid\left(Y_{hmb}\right)$$

(12) $$W_{1}=\;Y_{hmb}^{'}\times \left(\sum^{}Pool_{k\times k}\left(Y_{hmb}^{'}\right)\right)/3$$

where $X_{i-1}$ represents the output feature map of the i−1 -th convolutional layer, $Conv_{i}$ represents the i -th convolution, BN represents Batch Normalization, $Pool_{k\times k}$ represents the corresponding pooling layer and the k means the kernel size of pool layer. The size of the feature maps output by all pooling layers is the same as that of $Y_{hmb}^{'}$ .

To effectively enhances the network’s ability to capture the boundaries of the segmentation tissue, we introduce SEA to strengthen the features in the edge regions. Since the pixel values at the edges of the segmentation tissue change significantly, the information in these regions is more complex and uncertain, resulting in higher information entropy. Based on this, we calculate the information entropy of each pixel and assign higher weights to the pixels with higher information entropy, thereby emphasizing the regions with more complex and uncertain information. Assume $x_{p\times q}^{i}$ represents the feature at spatial position p × q in the i -th channel of $Y_{hmb}$ . SEA can be represented by Equations 13–15


(13) $$H\left(x_{p\times q}\right)=-\sum_{i=1}^{C}o\left(x_{p\times q}^{i}\right)\text{log}\left(o\left(x_{p\times q}^{i}\right)\right)$$

(14) $$W_{p\times q}=\frac{H\left(x_{p\times q}\right)}{\text{log}\left(C\right)}$$

(15) $$W_{2}=\{W_{p\times q}\;|\;p\in H,\;q\in W\}$$

where $H\left(x_{p\times q}\right)$ , $W_{p\times q}$ , and C represents the information entropy, spatial entropy weight and number of channels at p×q . H and W represent the height and width of the current feature map, respectively. Finally, we fuse $W_{1}$ and $W_{2}$ to obtain the final weight map, and perform element-wise multiplication on it with $Y_{hmb}$ . A residual connection is introduced to produce a more robust output feature map, $Y_{msb}$ . The above process can be expressed as Equation 16.


(16) $$Y_{msb}=\left(W_{1}+W_{2}\right)⊙Y_{hmb}+\;Y_{hmb}$$

## 4 Experiments

### 4.1 Datasets and evaluation metrics

We validated the performance of the proposed BE-Net in two MHSI segmentation tasks: gastric mucosal intestinal metaplasia (IM) and gastric intraepithelial neoplasia (GIN) (45). The IM dataset includes 412 microscopic hyperspectral pathological images. The image resolution is 512×512, with 40 spectral bands. The GIN dataset includes 282 microscopic hyperspectral pathological images. The image resolution is 512×512, with 40 spectral bands. Each image is annotated at the pixel level by pathology experts. To evaluate the performance of the model in segmentation tasks, we use overall accuracy (OA), Dice coefficient (DSC), Intersection over Union (IoU), Precision (Pre), Specificity (Spe), Sensitivity (Sen) and Standard deviation of the five-fold experiment as our evaluation metrics. These metrics allow for a comprehensive analysis of the model’s accuracy and effectiveness in handling different tasks. All experiments are conducted using five-fold cross-validation.

### 4.2 Experiments detail

The method implementation is carried out on a computer with an Intel i7-11700K, an NVIDIA GeForce RTX4090 GPU, RAM24GB, and 32GB of memory. We use SGD optimizer with batch size of 4. In IM, the initial learning rate is 0.01, while in GIN the initial learning rate is 0.008. In addition, we set the momentum to 0.9 and the weight decay to 0.000001, and the entire training process includes 80 epochs. We used the cross-entropy function and the Dice function as the model’s loss functions, with respective weights of 0.3 and 0.7. The number of PCA is 2. In each table presenting the experimental results, the highest accuracy achieved for each evaluation metric is highlighted in bold for easy reference.

### 4.3 Compared with other methods

In this work, we compare the proposed BE-Net with the current state-of-the-art methods, including U-net (4), Att-Unet (13), CA-Net (12), TransUnet (14) and MissFormer (15), to demonstrate the robustness and effectiveness of BE-Net. U-net is a classic encoder-decoder network widely used for image segmentation. Att-Unet designs an attention gate at the skip connections to help the model highlight regions in the image that are relevant to the segmentation task. CA-Net emphasizes the most important features through multi-dimensional attention mechanisms, enabling accurate segmentation of interest regions. TransUnet and MissFormer both implemented based on the self-attention mechanism. TransUnet employs CNNs to extract shallow features and enhances the model’s representation ability by combining local and global semantic information. MissFormer introduces an enhanced transformer context bridge to strengthen the connection between local and global hierarchical multi-scale features. All experimental training strategies are consistent with the proposed method. These methods have conducted in-depth studies in medical image segmentation, boundary detection, and multi-scale feature extraction. Their work is similar to our research focus and thus provides comparability.

#### 4.3.1 Experiment results on IM dataset

As shown in **Table 1**, BE-Net outperforms all comparison methods, achieving performance metrics of 94.14% for OA, 92.40% for DSC, 85.87% for IoU, 93.73% for Pre, 96.09% for Spe, and 91.13% for Sen. In terms of the aforementioned evaluation metrics, BE-Net outperforms the Unet by 1.4%, 1.68%, 2.85%, 2.05%, and 0.35%, respectively. Notably, BE-Net achieved an IoU score of 85.87%, representing a significant improvement over U-Net, which had an IoU score of 83.02%. Moreover, compared to the second-best method, Att-Unet, BE-Net achieved improvements of 0.83%, 1.02%, 1.74%, 1.55%, 1.02%, and 0.47% in OA, DSC, IoU, Pre, Spe, and Sen, respectively. Compared to the UNet, Att-Unet bridges the gap between the encoder and decoder through attention gates, further improving segmentation accuracy. On this basis, BE-Net further enhances the boundary feature representation ability of the encoder and decoder through LCB and MSB. The LCB module captures edge features, significantly improving the model’s ability to represent the shapes and spatial relationships of biological tissues, and the MSB module, located in the decoder, utilizes a multi-scale pyramid and spatial edge attention to better recover spatial details, leading to enhanced segmentation results. Additionally, BE-Net also designs GMB, which is similarly used to mitigate the semantic gap between the encoder and decoder. Through the combined effect of LCB, MSB, and GMB, BE-Net achieved the best segmentation results. CA-Net attempts to emphasize the most important features through a multi-dimensional attention mechanism. However, its performance on the IM dataset is lower than that of Att-Unet and BE-Net, which also capture important local discriminative features based on attention mechanisms. This may be because the model is lightweight, which representation capability is insufficient when faced with the complex feature distribution of MHSI, leading to inadequate focus on important regions and, consequently, lower segmentation performance. TransUnet and MISSFormer introduce the self-attention mechanism to capture global information in MHSI. From the **Table 1**, it can be seen that MISSFormer achieved the worst results among all the comparison methods. Compared to BE-Net, it is lower by 2.09% for OA, 2.62% for DSC, 4.36% for IoU, 3.58% for Pre, 2.32% for Spe, and 1.69% for Sen. This may be due to MISSFormer not considering the contribution of local features to the final segmentation accuracy, resulting in the worst performance. After incorporating CNN to extract local features, TransUnet outperforms MISSFormer by 1.17%, 1.5%, 2.47%, 1.61%, 1.01%, and 1.39% in OA, DSC, IoU, Pre, Spe, and Sen, respectively. However, it performs lower than BE-Net by 0.92%, 1.12%, 1.91%, 1.97%, 1.31%, and 0.3% in OA, DSC, IoU, Pre, Spe, and Sen, respectively. These results demonstrate that BE-Net achieved optimal performance in IM segmentation, further validating its effectiveness and superiority.

**Table 1:** Experiments on IM dataset (%).

Architecture	OA	DSC	IoU	Pre	Spe	Sen
U-Net	92.74 ± 0.62	90.72 ± 0.78	83.02 ± 1.31	90.73 ± 2.41	94.04 ± 1.60	90.78 ± 1.36
Att-Unet	93.31 ± 0.47	91.38 ± 0.64	84.13 ± 1.08	92.18 ± 2.31	95.07 ± 1.47	90.66 ± 1.45
CA-Net	92.94 ± 0.84	90.90 ± 1.17	83.34 ± 1.96	91.52 ± 1.91	94.63 ± 1.25	90.32 ± 1.32
TransUnet	93.22 ± 1.02	91.28 ± 1.33	83.98 ± 2.24	91.76 ± 2.17	94.78 ± 1.36	90.83 ± 1.46
MISSFormer	92.05 ± 1.40	89.78 ± 1.85	81.51 ± 3.03	90.15 ± 2.38	93.77 ± 1.37	89.44 ± 1.99
BE-Net	**94.14 ± 0.44**	**92.40 ± 0.65**	**85.87 ± 1.13**	**93.73 ± 1.56**	**96.09 ± 0.98**	**91.13 ± 1.03**

**Figure 6:**
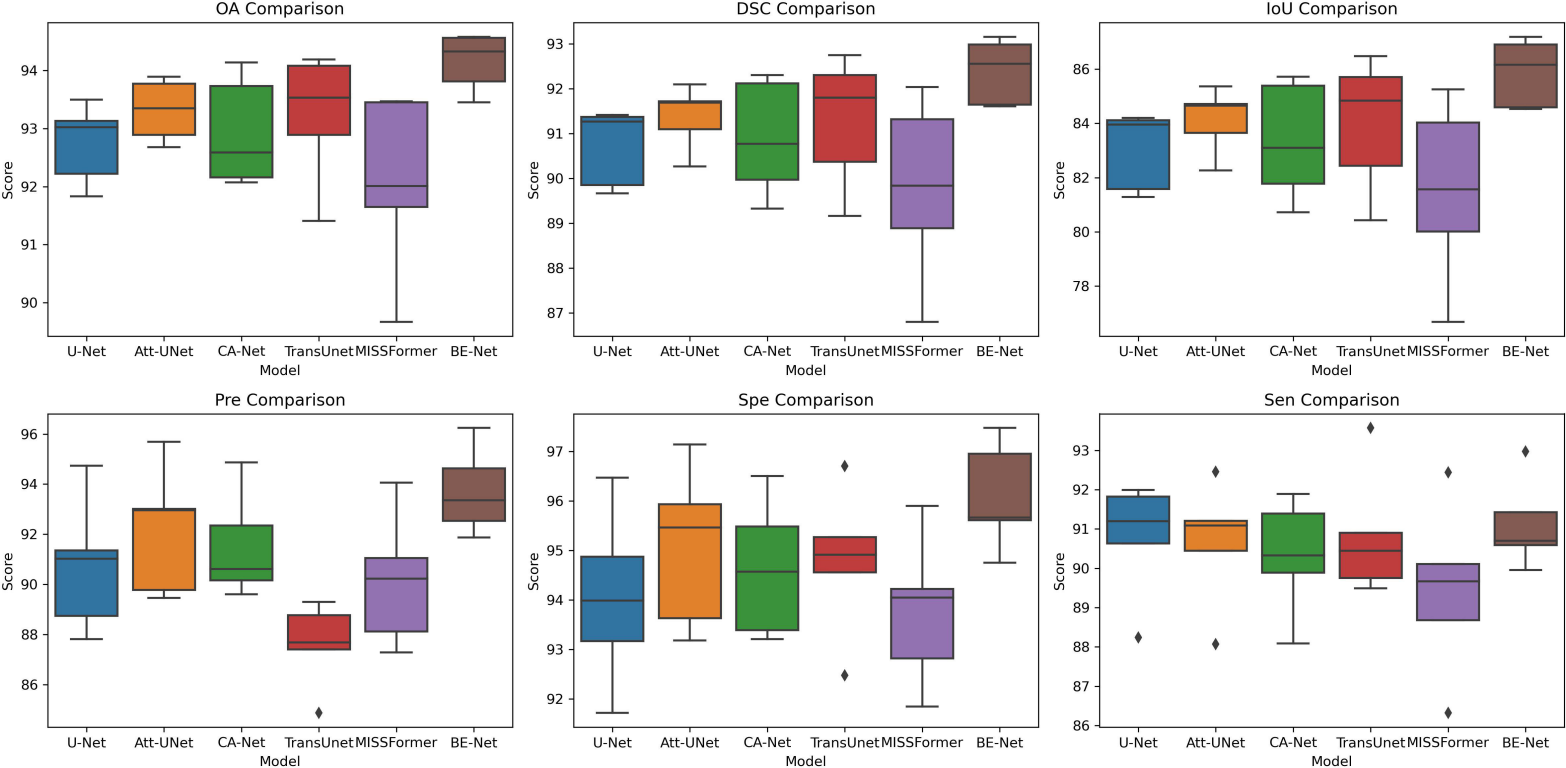
Box plot of the five-fold experiment on the IM dataset.

In **Figure 6**, we further present the results of five-fold cross-validation experiments for each model on the IM dataset, visualized using box plots, to provide a more intuitive comparison of the data across the five experiments. It can be seen that in terms of OA, DSC, IoU, Pre, and Spe, BE-Net not only demonstrates higher median values but also exhibits smaller variability, indicating that its performance is more consistent across five-fold experimental samples, with more stable and reliable results. In terms of Sen, although the median of BE-Net is slightly lower than that of U-Net and Att-UNet, both of these models exhibit small outliers in their Sen. This indicates that, in some fold, it may not be possible to detect target regions well. In summary, compared to other models, BE-Net demonstrates superior performance across all metrics.

**Figure 7:**
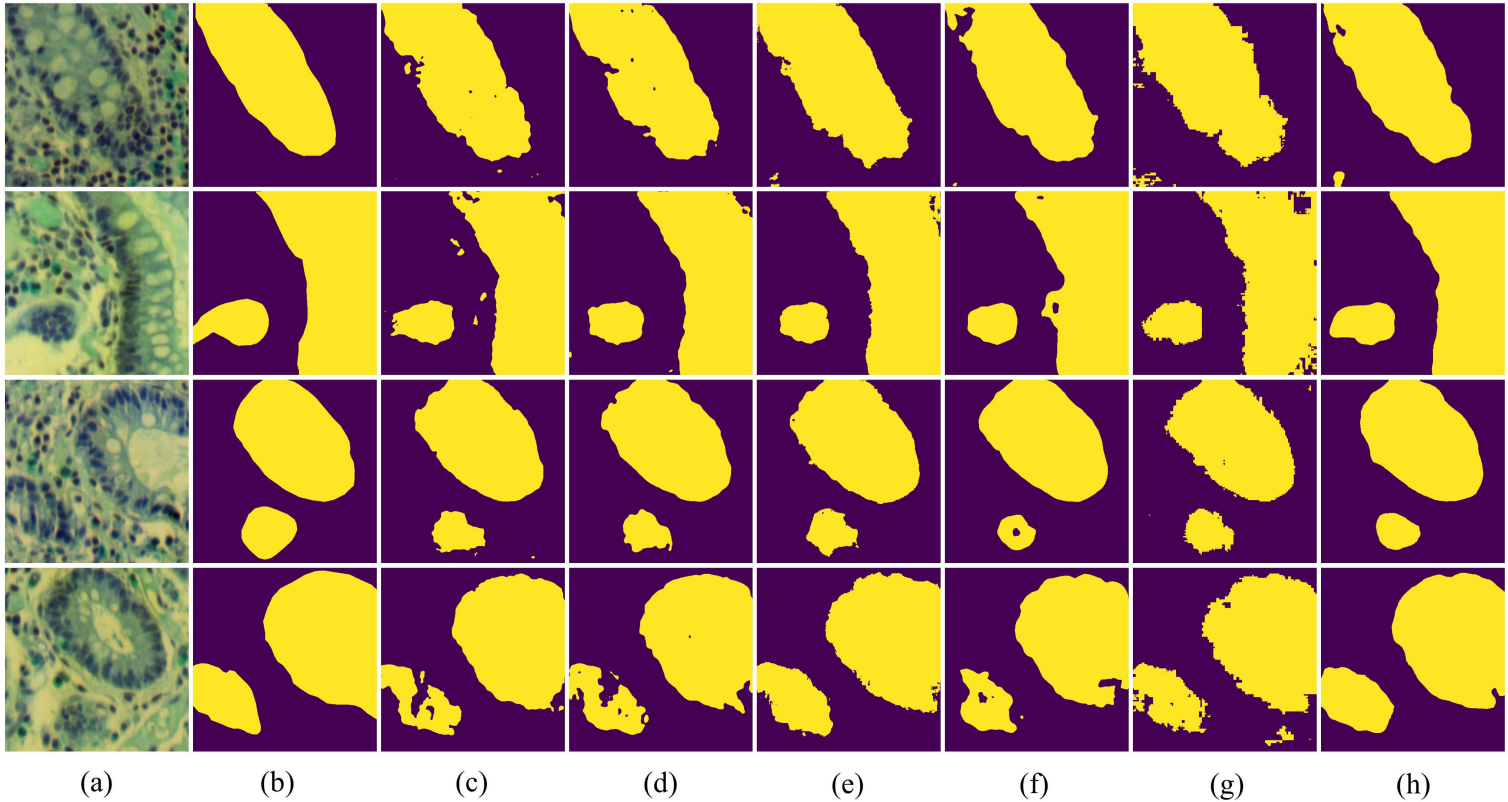
Vision comparison on IM dataset: **(a)** False color image of MHSIs;(b) Ground Truth; **(c)**Unet; **(d)** Att-Unet; **(e)** CA-Net; **(f)** TransUnet; **(g)** MissFormer; **(h)** BE-Net.

In **Figure 7**, we present the qualitative comparison results of different methods on the IM dataset. The visualization results of BE-Net are noticeably superior to the other methods. On one hand, the visualization of BE-Net is closer to the ground truth (GT), with significantly fewer missed detections and erroneous segmentation areas compared to other methods. On the other hand, its boundary regions are smoother and more complete, providing more accurate boundary delineation than other methods. This is because the proposed modules, especially the design of CEA and SEA, have enhanced the model’s ability to represent the edge features of biological tissues. In summary, compared to other methods, BE-Net produced more accurate segmentation.

#### 4.3.2 Experiment results on GIN dataset

We present the experimental results of the relevant methods on GIN in **Table 2**. From **Table 2**, it can be seen that the proposed method achieved 89.70%, 89.55%, 81.08%, 89.43%, 89.72%, and 89.71% for OA, DSC, IoU, Pre, Spe, and Sen, respectively, outperforming other methods in all metrics. Compared to the second-best method, Att-Unet, BE-Net improved by 1.37% and 2.21% in DSC and IoU, respectively. Same to the experiment on IM dataset, it can be observed that the segmentation accuracy of TransUnet and MissFormer is significantly lower than other networks. This may be attributed to the relatively small number of training samples in GIN dataset and the parameters in TransUnet and MissFormer are larger, which lead to overfitting and result in a decline in accuracy. It is worth mentioning that, on the GIN dataset, MISSFormer outperforms TransUnet by 0.1%, 0.22%, 0.33%, and 0.92% in OA, DSC, IoU, and Sen, respectively, in the absence of local feature learning. This may be because TransUnet has a larger number of parameters, requiring more data for training. Therefore, in the GIN dataset, TransUnet is prone to overfitting, which leads to a decrease in final segmentation accuracy.

**Table 2:** Experiments on GIN dataset (%).

Architecture	OA	DSC	IoU	Pre	Spe	Sen
U-Net	88.11 ± 0.43	88.07 ± 0.39	78.68 ± 0.62	87.04 ± 1.05	87.08 ± 1.39	89.14 ± 0.96
Att-Unet	88.31 ± 0.24	88.18 ± 0.42	78.87 ± 0.68	87.76 ± 1.53	88.01 ± 1.38	88.66 ± 1.66
CA-Net	87.10 ± 1.36	86.77 ± 1.58	76.67 ± 2.43	87.57 ± 2.15	88.16 ± 1.84	86.18 ± 4.09
TransUnet	85.73 ± 1.18	85.47 ± 1.26	74.65 ± 1.92	85.66 ± 1.62	86.15 ± 1.32	85.33 ± 1.84
MISSFormer	85.83 ± 1.13	85.69 ± 1.22	74.98 ± 1.90	85.19 ± 1.90	85.46 ± 1.78	86.25 ± 2.06
BE-Net	**89.70 ± 0.68**	**89.55 ± 0.75**	**81.08 ± 1.23**	**89.43 ± 2.06**	**89.72 ± 1.86**	**89.71 ± 0.71**

**Figure 8:**
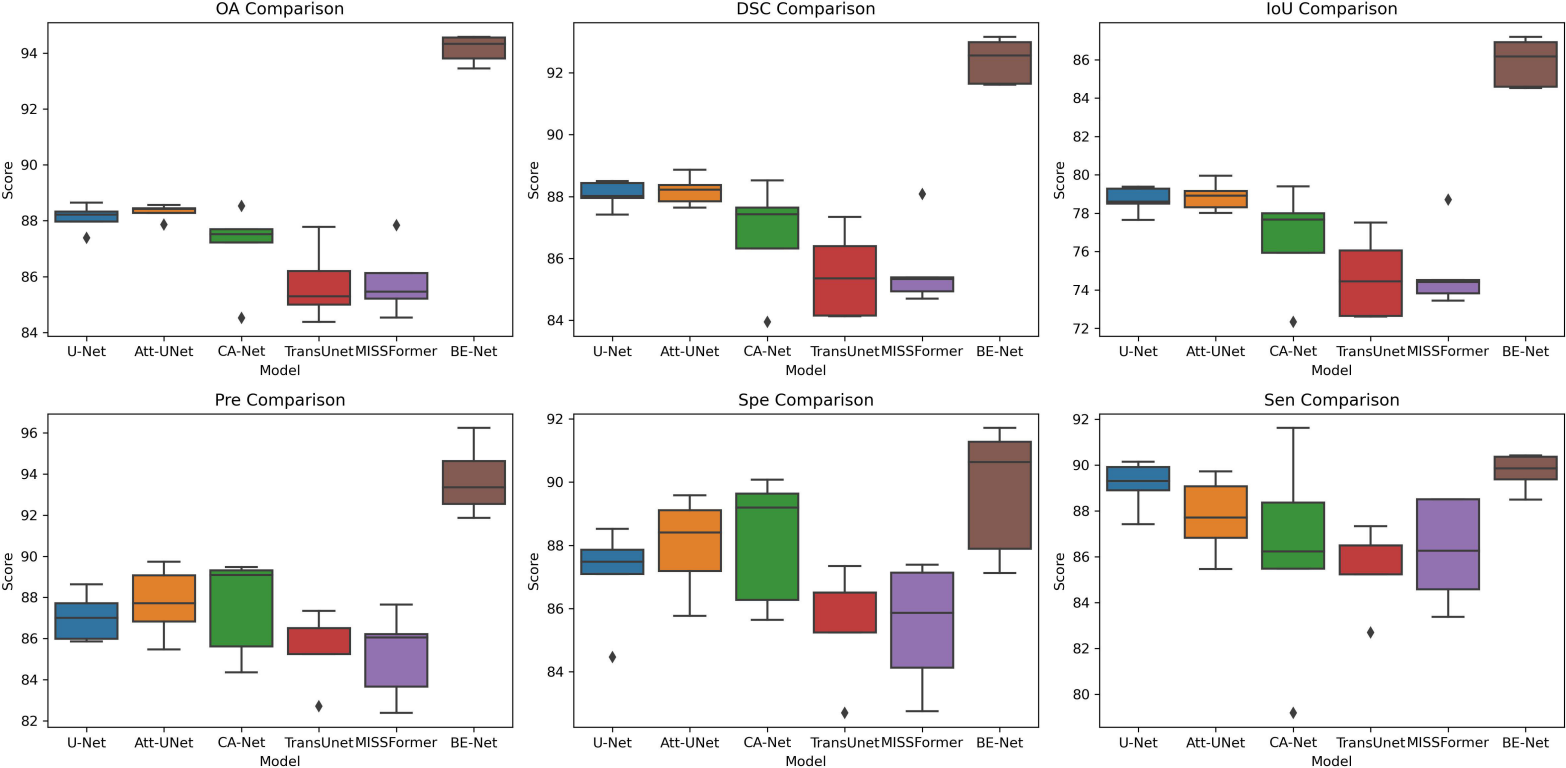
Box plot of the five-fold experiment on the GIN dataset.

In **Figure 8**, we further visualize the results of the five-fold cross-validation experiments for each model on the GIN dataset using box plots. From the **Figure 8**, it is evident that BE-Net performs outstanding across all metrics, particularly in OA, DSC, IoU, and Sen, where it shows high median values and smaller variability. This means that BE-Net demonstrates higher robustness in MHSI segmentation tasks compared to other comparative models. Compared with other methods, the better performance in IoU and Sen indicates that BE-Net can accurately and stably segment key regions, such as tumors or lesion areas.

**Figure 9:**
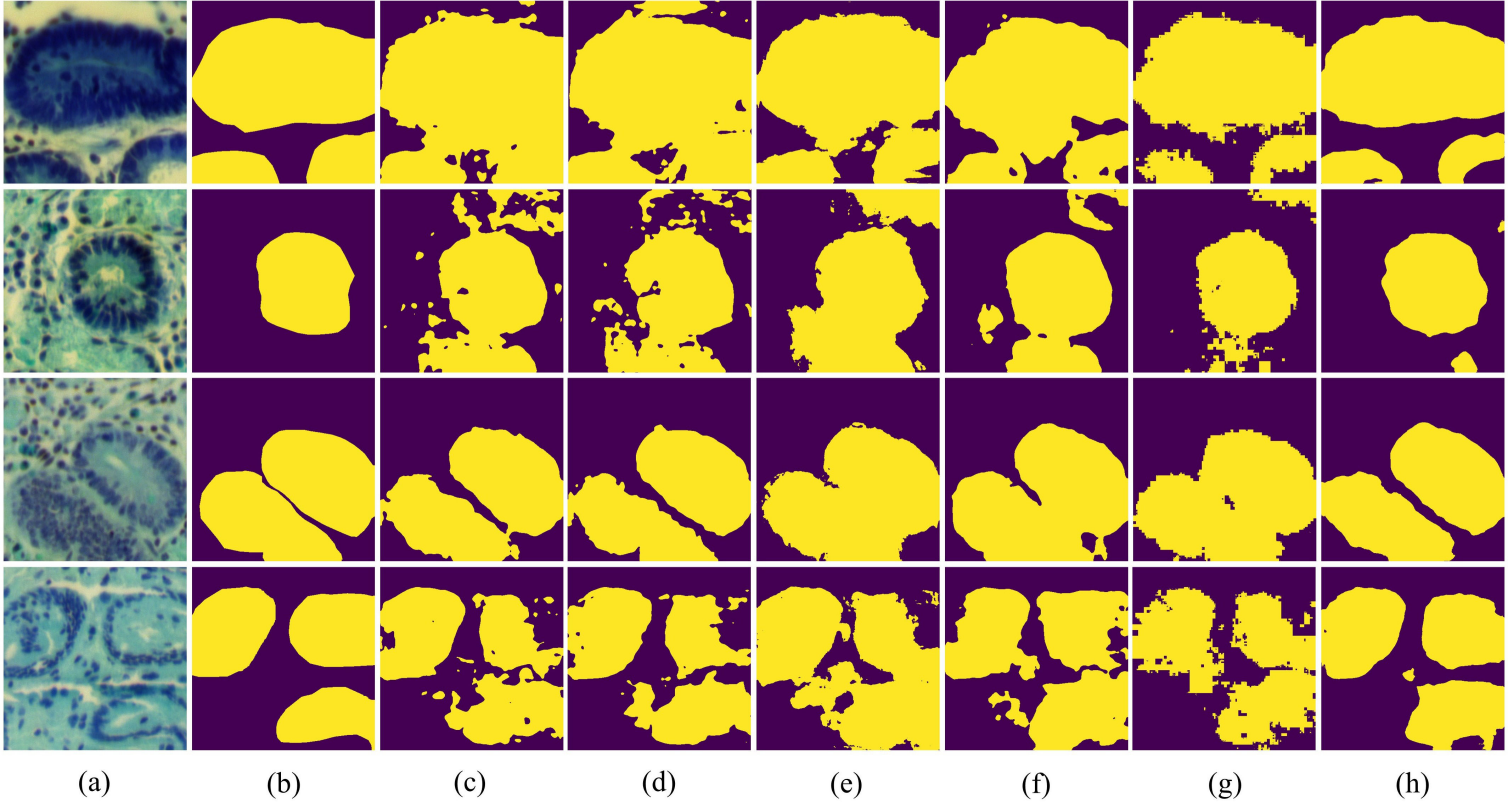
Vision comparison on GIN dataset: **(a)** False color image of MHSIs;(b) Ground Truth; **(c)**Unet; **(d)** Att-Unet; **(e)** CA-Net; **(f)** TransUnet; **(g)** MissFormer; **(h)** BE-Net.

In **Figure 9**, we present the visualization results of all experiments on the GIN dataset. It can be seen that the proposed BE-Net has the fewest misclassification areas compared to other networks. Furthermore, BE-Net exhibits more precise segmentation boundaries across regions of varying sizes and shapes compared to other methods. This indicates that BE-Net, leveraging the proposed LCB, GMB, and MSB modules, captured more detailed information about the segmentation tissues, demonstrating better capability in learning discriminative boundary features. In summary, the proposed BE-Net achieved the best segmentation performance among all the compared methods on GIN dataset.

#### 4.3.3 Comparison of computation time and complexity on IM dataset

We present the computation time and computational complexity of all models on the IM dataset in **Table 3**. As shown in **Table 3**, the number of parameters in BE-Net is only 3.757M, significantly lower than that of all other models except CA-Net, particularly UNet and Att-UNet. This means that BE-Net achieves high performance while maintaining a smaller model size, thus reducing the demand for storage and computational resources. Although CA-Net has fewer parameters, its performance in both quantitative analysis and visualization is poor. BE-Net’s FLOPs are 29.606G, significantly lower than those of other models. This means that BE-Net is more computationally efficient. In terms of training time, BE-Net’s training time is 2047.19 seconds, which is relatively shorter compared to other models. Especially when compared to UNet and Att-UNet, which have better segmentation performance, BE-Net reduces training time by 1061.87 and 1306.9 seconds, respectively, demonstrating its efficiency in model training. In terms of testing time, BE-Net’s testing time is 7.99 seconds, which is close to that of TransUnet and MISSFormer, indicating its relatively fast speed during the inference phase. Overall, BE-Net excels in terms of parameter count, computational complexity, training time, and testing time. Compared to other methods, its efficiency and high performance offer significant advantages in MHSIs segmentation.

**Table 3:** Comparison of computation time and complexity on IM dataset.

Architecture	Paras	FLOPs	Trian time (s)	Test time (s)
U-Net	34.53M	1.05T	3109.06	10.83
Att-Unet	34.88M	1.07T	3354.09	9.03
CA-Net	**2.79M**	95.65G	2222.31	8.48
TransUnet	100.90M	276.06G	**1957.50**	**7.59**
MISSFormer	35.45M	147.85G	2500.72	7.95
BE-Net	3.757M	**29.606G**	2047.19	7.99

### 4.4 Ablation experiment

#### 4.4.1 Comparison of the effect of combining different modules

To validate the effectiveness of each component in the proposed BE-Net, we conducted a series of experiments using the IM dataset as an example. These experiments evaluated the performance of different models formed by combining various components, including the proposed LCB, GMB, and MSB. In these experiments, we used a baseline network with channel numbers at each level set to one-quarter of the original as the U-net. The proposed modules were then gradually integrated into the baseline model.

**Table 4** presents the results of the ablation experiments for different component combinations. It can be seen that, compared to the baseline, the addition of the proposed LCB, GMB, and MSB resulted in varying degrees of improvement in the model’s performance. In Experiment 2, the addition of LCB improved the baseline results by 0.83%, 1.05%, 1.76%, 0.9%, 0.58%, and 1.15% in OA, DSC, IoU, Pre, Spe, and Sen, respectively. This is because LCB extracts gradient information between features through LoGC, greatly enriching the edge information of tissues. Additionally, the inclusion of CEA emphasizes channel features which relevant to the segmentation task while suppressing redundant information. Furthermore, when both the LCB and GMB modules were incorporated, the model exhibited improvements across all six metrics. Specifically, for the IoU metric, the addition of both LCB and GMB resulted in an improvement of 1.61% compared to the result achieved with only LCB. This validates that the proposed GMB, through GMB and CEA, further optimized feature representation and mitigated the semantic gap between deep and shallow features. In Experiment 4, the addition of LCB, GMB and MSB, namely BE-Net achieved the best results. Compared to Experiment 3, it improved by 0.28%, 0.46%, 0.79%, 0.7%, 0.32%, and 0.21% in OA, DSC, IoU, Pre, Spe, and Sen, respectively. This proves that MSB can further optimize spatial features.

**Table 4:** Ablation study on the proposed components of the BE-Net with the IM dataset.

No.	LCB	GMB	MSB	OA	DSC	IoU	Pre	Spe	Sen
1	×	×	×	92.18 ± 0.57	89.93 ± 0.70	81.71 ± 1.14	90.64 ± 2.64	94.06 ± 1.81	89.32 ± 1.64
2	✓	×	×	93.01 ± 0.80	90.98 ± 1.21	83.47 ± 2.05	91.54 ± 2.12	94.64 ± 1.31	90.47 ± 1.70
3	✓	✓	×	93.86 ± 0.45	91.94 ± 0.78	85.08 ± 1.34	93.03 ± 1.95	95.77 ± 1.07	90.92 ± 1.52
4	✓	✓	✓	**94.14 ± 0.44**	**92.40 ± 0.65**	**85.87 ± 1.13**	**93.73 ± 0.98**	**96.09 ± 0.98**	**91.13 ± 1.03**

In the table, “✓” indicates that the module is used, while “×” denotes that the module is not included.

**Figure 10:**
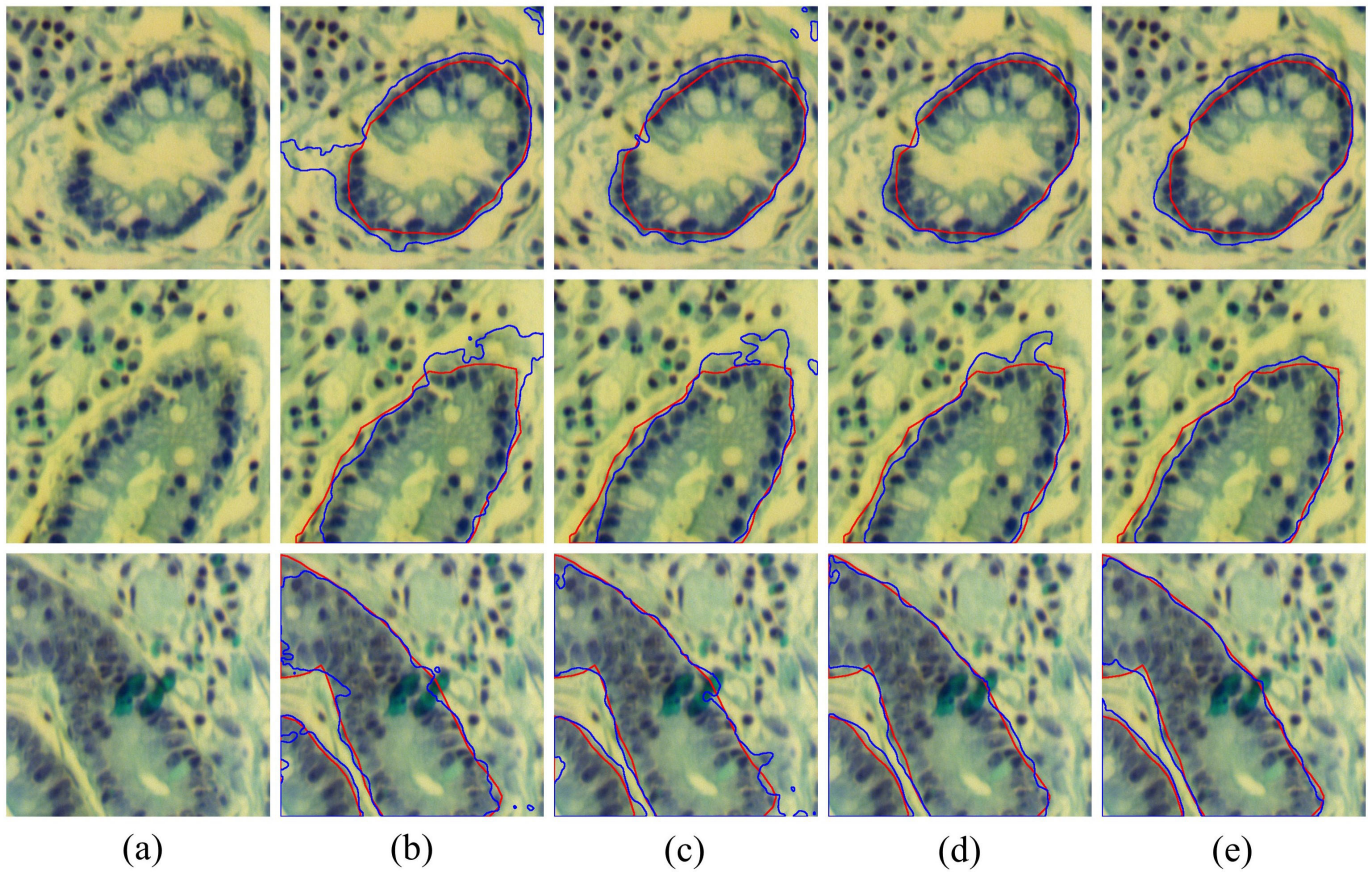
Visualization results of combining different modules, the red contours represent the ground truth, and bule contours represent the predicted segmentation: **(a)** False color image of MHSIs; **(b)** Baseline; **(c)** Baseline+LCB; **(d)** Baseline+LCB+GMB; **(e)** Baseline+LCB+GMB+MSB.

To further validate the effectiveness of each module, we present several common challenges in **Figure 10**, including boundary fractures (the first row), boundary blurring (the second row), and external interference (the third row), along with the visualized boundary predictions of each model under these challenging conditions. It can be seen that the baseline results are the worst, as it neglects the extraction of boundary information, often leading to over-segmentation or under-segmentation boundary. The results of Baseline + LCB are better than the baseline. Notably, when handling broken edges and blurred boundaries, the issue of over-segmentation is significantly improved. By combining LCB and GMB, the model’s accuracy in boundary segmentation was further improved, indicating that the proposed GMB effectively enhances the model’s ability to perceive and delineate tissue boundaries. The fifth column of **Figure 10** shows the segmentation results of BE-Net, which are the closest to the ground truth. Compared to other models which exhibit over segmentation and under segmentation at points of broken edges, blurring, and interference, BE-Net shows significant improvement. In summary, each module in the proposed method contributes positively to the final results, and when all three are combined, the best segmentation performance is achieved.

#### 4.4.2 Ablation experiment for the information entropy weight in the LCB, GMB and MSB modules

In BE-Net, we designed CEA and SEA based on information entropy to comprehensively analyze features, thereby emphasizing important features and suppressing irrelevant redundant information. To validate the effectiveness of CEA and SEA, we performed ablation studies in LCB, GMB, MSB. Specifically, we compared the performance of LCB, GMB, and MSB (with CEA and SEA by default) with their performance when CEA and SEA were not used. **Tables 5**–**7** present the quantitative comparisons from the relevant ablation experiments, where ‘w/o’ indicates the absence of entropy weighting. These ablation results confirm the effectiveness of the proposed SEA and CEA. It should be pointed out that the addition of CEA in GMB resulted in a decrease in Sen. Considering that the inclusion of CEA improved the model’s performance in other five indicators, especially on locating target tissues and overall classification accuracy, it can be concluded that the integration of CEA in the GMB module is also beneficial. Based on the above analysis, it can be seen that the inclusion of CEA and SEA further enhances the model’s performance. Both make a positive contribution to the final segmentation performance of MHSIs.

**Table 5:** Segmentation results of the proposed LCB with and without the CEA (%).

Methods	OA	DSC	IoU	Pre	Spe	Sen
Baseline+LCB(w/o)	92.54 ± 0.56	0.44 ± 0.68	82.56 ± 1.12	90.66 ± 2.67	94.03 ± 1.73	90.32 ± 1.43
Baseline+LCB	**93.01 ± 0.80**	**90.98 ± 1.21**	**83.47 ± 2.05**	**91.54 ± 2.12**	**94.64 ± 1.31**	**90.47 ± 1.70**

**Table 6:** Segmentation results of the proposed GMB with and without the CEA (%).

Methods	OA	DSC	IoU	Pre	Spe	Sen
Baseline+GMB(w/o)	92.92 ± 0.61	91.03 ± 0.85	83.27 ± 1.34	91.65 ± 2.63	94.73 ± 1.67	**90.18 ± 1.71**
Baseline+ GMB	**93.41 ± 0.43**	**91.34 ± 0.71**	**84.07 ± 1.20**	**93.83 ± 1.31**	**96.27 ± 0.69**	89.00 ± 1.18

**Table 7:** Segmentation results of the proposed MSB with and without the SEA (%).

Methods	OA	DSC	IoU	Pre	Spe	Sen
Baseline+MSB(w/o)	92.95 ± 0.62	90.91 ± 0.77	83.34 ± 1.29	91.71 ± 2.02	94.77 ± 1.29	90.16 ± 0.93
Baseline+ MSB	**93.08 ± 0.68**	**91.08 ± 0.90**	**83.64 ± 1.51**	**91.75 ± 2.45**	**94.79 ± 1.53**	**90.50 ± 1.39**

#### 4.4.3 Ablation experiment for the other propose modules in LCB

To validate the rationality of combining the proposed LoG operator with Conv, we introduced the Prewitt operator, Sobel operator, and standard convolution for comparison with LoG. The horizontal and vertical templates of the Prewitt operator are $g_{x}\;=\;\left[\left[-1,\;0,\;1],\;[-1,\;0,\;1],\;[-1,\;0,\;1\right]\right]\;$ and $g_{y}\;=\;\left[\left[1,\;1,\;1],\;[0,\;0,\;0],\;[-1,-1,-1\right]\right]$ , respectively. The horizontal and vertical templates of the Sobel operator are $g_{x}\;=\;\left[\left[-1,\;0,\;1],\;[-2,\;0,\;2],\;[-1,\;0,\;1\right]\right]$ and $g_{y}=\;\left[\left[1,\;2,\;1],\;[0,\;0,\;0],\;[-1,-2,-1\right]\right]$ , respectively. Then, we approximate the gradient magnitude by using the sum of the absolute values of the horizontal and vertical components, expressed by the formula $G\left(x,\;y\right)\;=\;\left|g_{x}\left|\;+\;\right|g_{y}\right|$ . Finally, we replaced the LoG operator with the aforementioned operators, and the experimental results are shown in **Table 8**. It can be seen that, compared to using standard convolution for feature extraction, the method incorporating edge detection operators achieved improvements across all six-evaluation metrics. In addition, compared to the Prewitt and Sobel operators, LoG achieved the best results in terms of OA, DSC, and IoU.

**Table 8:** Experiment results of the proposed LCB with different module (%).

Methods	OA	DSC	IoU	Pre	Spe	Sen
Baseline	92.18 ± 0.57	89.93 ± 0.70	81.71 ± 1.14	90.64 ± 2.64	94.06 ± 1.81	89.32 ± 1.64
Baseline+LCB(Conv)	92.71 ± 0.44	90.63 ± 0.80	82.87 ± 1.33	90.87 ± 1.44	94.18 ± 0.86	90.42 ± 1.24
Baseline+LCB(prewitt)	92.63 ± 0.98	90.64 ± 1.26	82.91 ± 2.10	90.12 ± 2.84	93.58 ± 1.85	**91.24 ± 1.39**
Baseline+LCB(sobel)	92.70 ± 0.63	90.56 ± 0.77	82.76 ± 1.27	**91.62 ± 2.79**	**94.73 ± 1.77**	89.62 ± 1.64
Baseline+LCB(LoGC)	**93.01 ± 0.80**	**90.98 ± 1.21**	**83.47 ± 2.05**	91.54 ± 2.12	94.64 ± 1.31	90.47 ± 1.70

### 4.5 PCA experiment

**Figure 11:**
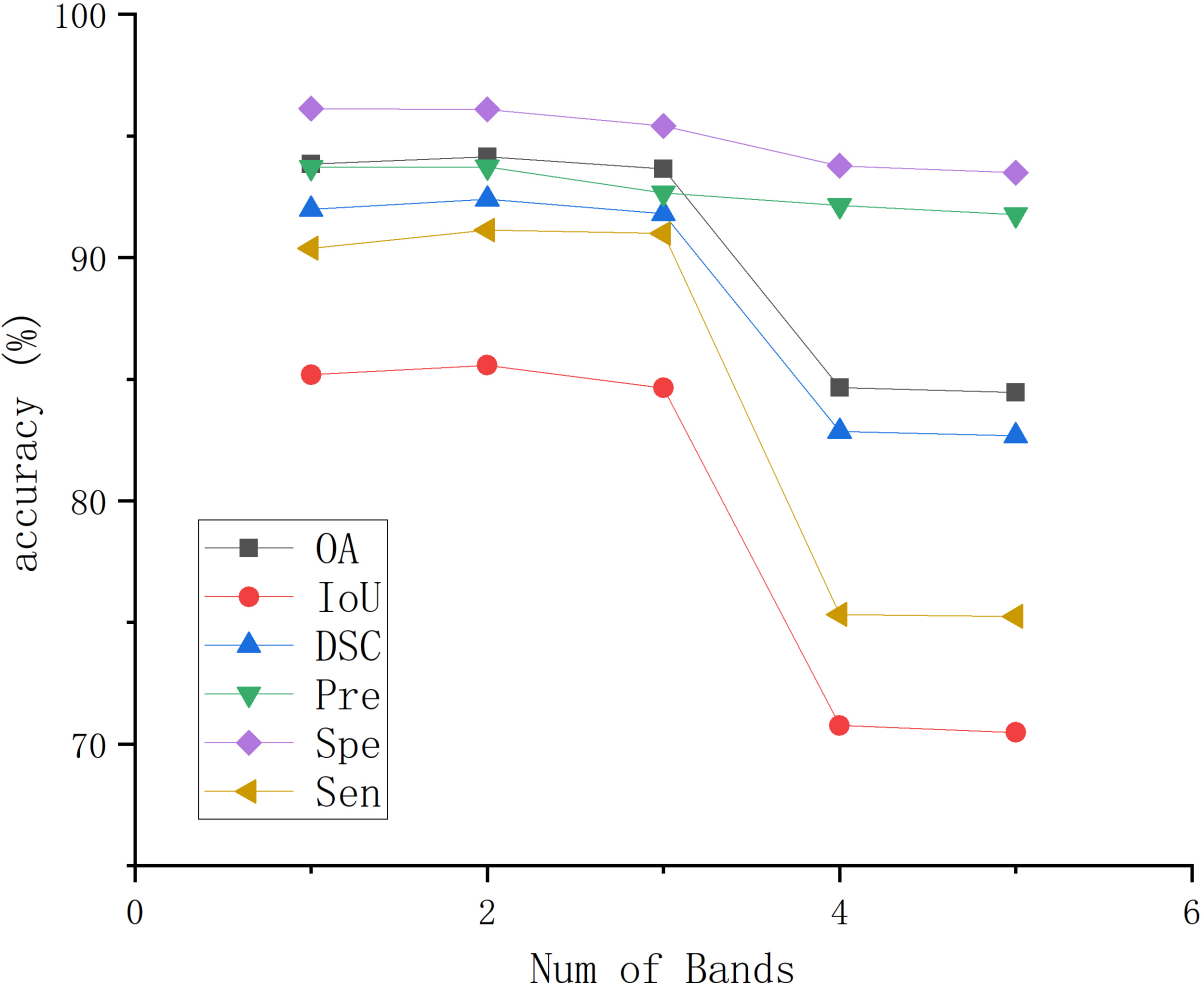
The effect of PCA on segmentation results on IM.

**Figure 12:**
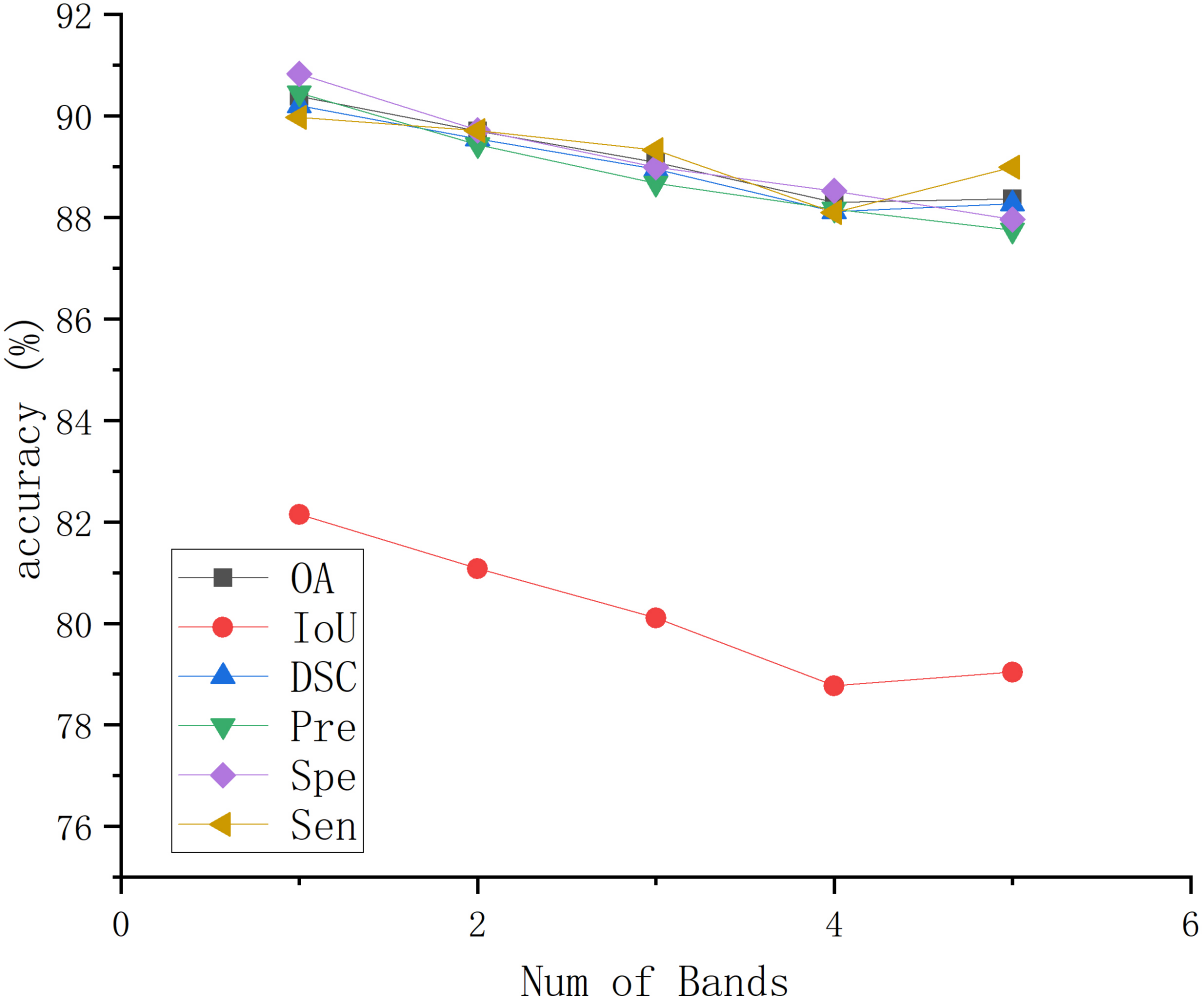
The effect of PCA on segmentation results on GIN.

In MHSIs segmentation tasks, researchers commonly apply PCA for dimensionality reduction to mitigate the impact of high correlation and similarity between spectral bands. To describe the choice of the selected PCA dimensions, we present the impact of PCA on the segmentation results for the IM and GIN datasets in **Figures 11**, **12**, respectively. In the figures, experiment 1 to 5 correspond to the first 1 to 5 spectral bands selected after applying PCA.

As shown in **Figure 11**, the experimental results of BE-Net on the IM dataset exhibit a trend of first increasing, then decreasing, and finally leveling off as the PCA dimensions increase. This is because, as the PCA dimensions increase, while more feature information is provided, many redundant features and noise are also introduced. This additional information can interfere with the model during training, leading to a decline in performance. In **Figure 12**, it can be observed that as the number of retained PCA dimensions increases, the experimental results of BE-Net on the GIN dataset exhibit a trend of first decreasing and then increasing. This is because the GIN dataset is relatively small, causing the model to heavily rely on the detailed information in the training data. On one hand, this can lead to overfitting, while on the other hand, noise or irrelevant features may also affect its performance. As the PCA dimensions are further increased, more data features and complex patterns in the MHSIs are retained, allowing BE-Net to better capture the underlying structure within the data, thus showing an upward trend. Based on the above analysis, and after careful consideration, this study retains first 2 spectral bands after PCA.

## 5 Conclusion

In MHSIs, the complex structures and boundaries of tissues present a significant challenge for accurate segmentation. To alleviate this issue, this paper combines edge detection operators and multi-scale extraction strategies with an information entropy-based attention mechanism to further optimize feature representation. This new information entropy-based attention mechanism is of great significance for improving the segmentation accuracy of MHSIs.

Specifically, we designed a boundary aware segmentation network guided by information entropy weight (BE-Net) to achieve more accurate segmentation results in MHSI segmentation tasks. In BE-Net, we first developed a LCB to guide the model in focusing on edge channel feature information relevant to the segmentation task. Subsequently, we designed a GMB to alleviate the semantic gap between the encoder and decoder. This block further enriches the representation of boundary features while suppressing interference and redundancy information. Finally, we designed an MSB. This module guides the network to extract spatial pixel relationships in the decoder and emphasizes edge information to enable the network to achieve better boundary segmentation. Experiments on two MHSIs datasets demonstrate that the proposed method outperforms other advanced methods.

In future work, we will first further explore the application of information entropy in MHSI segmentation tasks to better analyze spatial and spectral features. Specifically, we will explore the fusion of multi-scale spatial-spectral features by introducing joint entropy, further investigating the role of entropy in a multi-scale context. At the same time, we will quantify the model’s prediction results in MHSIs based on information entropy, to improve the reliability of the model in complex MHSIs segmentation tasks. Secondly, due to the challenges of MHSI labeling and sampling, the total sample size in this paper’s dataset is relatively small. To further validate the generalizability of the proposed method, in future work, we will include MHSI labeling tasks and expand the dataset with more diverse cancer types to validate the relevant methods, further assessing their advancement and superiority.

## Data Availability

Publicly available datasets were analyzed in this study. This data can be found here: https://onlinelibrary.wiley.com/doi/10.1002/jbio.202200163.
